# Draft Genome Sequences of *Sphingomonas mucosissima* DSM 17494 and *Sphingomonas dokdonensis* DSM 21029

**DOI:** 10.1128/genomeA.00889-17

**Published:** 2017-08-31

**Authors:** Anja Poehlein, Jan Hendrik Wübbeler, Rolf Daniel, Alexander Steinbüchel

**Affiliations:** a Department of Genomic and Applied Microbiology and Göttingen Genomics Laboratory, Institut für Mikrobiologie & Genetik, Georg-August-Universität Göttingen, Göttingen, Germany; b Institut für Molekulare Mikrobiologie & Biotechnologie, Westfälische Wilhelms-Universität Münster, Münster, Germany; c Environmental Sciences Department, Faculty of Meteorology, Environment and Arid Land Agriculture, King Abdulaziz University, Jeddah, Saudi Arabia

## Abstract

*Sphingomonas mucosissima* and *Sphingomonas dokdonensis* are Gram-negative chemoheterotrophic strictly aerobic rods or cocci. The genomes (3.453 Mb and 3.587 Mb, respectively) contain 3,279 and 3,329 predicted protein-encoding genes, respectively. The genome of *S. dokdonensis* harbors a 90-kb plasmid.

## GENOME ANNOUNCEMENT

The genus *Sphingomonas* was proposed in 1990 (1), and since then, the scientific interest for sphingomonads, and thus the increasing count of publications for this novel species, are constantly rising (compare at http://www.bacterio.net/sphingomonas.html). Members of this genus are Gram-negative chemoheterotrophic strictly aerobic rods or cocci (1). The type species is *Sphingomonas paucimobilis* DSM 1098 (2, 3). The most characteristic feature of this genus is the presence of sphingolipids in their outer cell membrane (1, 4). During a study for the optimization of triacylglycerol synthesis in novel oleaginous bacteria (5), *Sphingomonas jeddahensis* G39^T^ was enriched and isolated from desert soil and published as a new species of the genus *Sphingomonas* (6). Its closest relative type strains are *Sphingomonas dokdonensis* DSM 21029 and *Sphingomonas mucosissima* DSM 17494. *S. dokdonensis* DSM 21029 was derived from Dokdo, a South Korean island with restricted human access (7). *S. mucosissima* DSM 17494 was isolated from the Colorado Plateau (USA) (8). In this study, both strains were sequenced, and the draft genomes were analyzed and are presented here.

Chromosomal DNA was isolated using the MasterPure complete DNA purification kit (Epicentre, Madison, WI, USA). The extracted DNA was used to generate Illumina shotgun paired-end sequencing libraries, which were sequenced with a MiSeq instrument and the MiSeq reagent kit (version 3), as recommended by the manufacturer (Illumina, San Diego, CA, USA). Quality filtering using Trimmomatic version 0.36 (9) resulted in 2,754,576 and 2,741,920 paired-end reads for *S. dokdonensis* and *S. mucosissima*, respectively. The assembly performed with the SPAdes genome assembler software version 3.10.0 (10) resulted in 16 contigs (>500 bp) for both genomes, with average coverages of 153-fold (*S. dokdonensis*) and 149-fold (*S. mucosissima*). The assembly was validated and the read coverage determined with QualiMap version 2.1 (11). The draft genome of *S. dokdonensis* DSM 21029 (3.453 Mb) exhibited an overall G+C content of 66.46%. The genome of *S. mucosissima* DSM 17494 (3.587 Mb), with an overall G+C content of 65.08%, is slightly larger than that of *S. dokdonensis*. Automatic gene prediction and identification of rRNA and tRNA genes were performed using the software tool Prokka (12). The draft genomes of *S. dokdonensis* and *S. mucosissima* contain 3,279 predicted protein-encoding and 54 RNA genes and 3,329 predicted protein-encoding and 52 RNA genes, respectively. The genome of *S. dokdonensis* putatively harbors a 90-kb plasmid, as genes encoding a RepB family protein, a partitioning protein ParA, and a replication protein B were located in a single contig. Genes encoding a serine palmitoyltransferase and an NADPH-dependent 3-ketodihydrosphingosine reductase, catalyzing the initial steps of *de novo* sphingolipid biosynthesis, have been identified in both genomes (13).

### Accession number(s).

These whole-genome shotgun projects have been deposited at DDBJ/EMBL/GenBank under the accession numbers NBBI00000000 and NBBJ00000000 for *S. dokdonensis* DSM 21029 and *S. mucosissima* DSM 17494, respectively. The versions described here are the first versions, NBBI01000000 and NBBJ01000000, respectively.
